# *Daboia (Vipera) palaestinae* Envenomation in 123 Horses: Treatment and Efficacy of Antivenom Administration

**DOI:** 10.3390/toxins11030168

**Published:** 2019-03-19

**Authors:** Sharon Tirosh-Levy, Reut Solomovich-Manor, Judith Comte, Israel Nissan, Gila A. Sutton, Annie Gabay, Emanuel Gazit, Amir Steinman

**Affiliations:** 1 Koret School of Veterinary Medicine, The Robert H. Smith Faculty of Agriculture, Food and Environment, The Hebrew University of Jerusalem, Rehovot 7610001, Israel; reutsolo@gmail.com (R.S.-M.); Judith.comte@mail.huji.ac.il (J.C.); gila.sutton@mail.huji.ac.il (G.A.S.); amirst@savion.huji.ac.il (A.S.); 2 Ministry of Health Central Laboratories, Jerusalem 9134302, Israel; israel.nissan@gmail.com (I.N.); anniegabay@gmail.com (A.G.); emgaz785@gmail.com (E.G.)

**Keywords:** *Vipera palaestinae*, *Daboia palaestinae*, envenomation, snakebite, horse, antivenom

## Abstract

Envenomation by venomous snakes is life threatening for horses. However, the efficacy of available treatments for this occurrence, in horses, has not yet been adequately determined. The aim of this study was to describe the treatments provided in cases of *Daboia palaestinae* envenomation in horses and to evaluate the safety and efficacy of antivenom administration. Data regarding 123 equine snakebite cases were collected over four years from 25 veterinarians. The majority of horses were treated with procaine-penicillin (92.7%), non-steroidal anti-inflammatory drugs (82.3%), dexamethasone (81.4%), tetanus toxoid (91.1%) and antivenom (65.3%). The time interval between treatment and either cessation or 50% reduction of local swelling was linearly associated with case fatality (*p* < 0.001). The overall mortality rate was 20.3%. Treatment with procaine-penicillin was significantly associated with reduced mortality (OR = 0.11). Three horse-derived antivenom products were available during the study period, of which the horses were administered different brands of varying dosages. Administration of the recommended dosage of any of the aforementioned products led to a significant decrease in mortality (*p* = 0.014), even in severe cases (scoring 2 or greater on the equine snakebite severity scale). No adverse reactions were reported. The results of this study show that species-specific *D. palaestinae* antivenom administered at the manufacturer-recommended dosage is effective in significantly reducing mortality in cases of envenomation in horses.

## 1. Introduction

Envenomation by venomous snakes is a major cause of morbidity and mortality in both humans and animals worldwide [1]. *Daboia palaestinae* (formerly: *Vipera (xanthine) palaestinae*, subfamily: *Viperinae*, true vipers), which is endemic to the Mediterranean area, including western Syria, northwestern Jordan, northern and central Israel, Palestine and Lebanon, is a leading cause of snakebites within its geographic range [2,3,4]. Incidences of envenomation by *D. palaestinae* were reported in humans, dogs [5,6,7], cats [8], horses [9] and a ram [10].

Treatment of *D. palaestinae* envenomation differs depending on the affected species and includes supportive measures, pain management, antibiotics, antihistamines and steroids, some of which are controversial within the scientific community [5,6,8,11,12,13]. The mainstay of treatment in human patients is prompt administration of antivenom, which is considered to be the only specific and effective treatment, reducing mortality from 6–10% to 0.5–2% [2,14,15,16]. The production and use of antivenom commenced more than a century ago, in 1897 [17]. Since then, species-specific antivenoms, are used routinely. Most antivenoms are produced from animal sera, most commonly horses’, which undergo a process of fractionation and purification to produce either whole IgG, F(ab’)2 or Fab fragments [17,18]. The differences in efficacy and safety of these different types have not been fully elucidated. However, generally speaking, it seems that the purity (or rather the impurity) of the product is the main factor influencing adverse reactions [17]. Horse-derived IgG monovalent *D. Palaestinae*-specific antivenom has been produced and used in Israel since the 1960s [19,20,21]. Initial dosage of 50 mL (five ten-mL vials) is usually effective in the treatment of a systemic disease in human, and the dosage may be increased in unresponsive cases [2,11,13,15]. Adverse reactions and serum sickness have been documented in humans [2,11,15,22]. Treatment of dogs and cats envenomed by *D. palaestinae* has been described, but efficacy of antivenom treatment has not been established [3,5,8]. No previous documentation exists regarding the treatment of horses for *D. palaestinae* envenomation. The use of antivenom in horses following rattlesnake or elapid snake envenomation seems to have been favorable, although conclusions regarding its efficacy have been limited [23,24].

In recent years, there has been a global effort to standardize the production of antivenom, and the Global Snakebite Initiative is attempting to develop a new polyvalent antivenom against the most common venomous snake species of Asia and Africa [17,25]. As part of this strategy, since 2011, *D. palaestinae* antiserum has been manufactured by Kamada Ltd. (Rehovot, Israel), a privately-owned company, in order to provide new polyvalent antivenom at good manufacturing practice (GMP) standards and to improve its safety. As a result, in 2013, there was a fivefold increase in the price of antivenom ($1400 US for one vial), which made it less affordable to veterinary patients, and led to the importation of two alternative veterinary products. At the time of this study, three products were available, which differ in their composition and concentration (Table 1). The main product that was imported (Viper-stat^®^, BioVeteria, Mexico City, Mexico) is a new product, which contains antivenom against several snake species from North Africa and the Middle East. The second is European viper venom antiserum, which is not specific for *D. palaestinae*, but is active against the closely related species *Vipera xantinae.*

The aims of this study were to describe the treatments administered in cases of *D. palaestinae* envenomation in horses and to evaluate the safety and efficacy of antivenom administration.

## 2. Results

### 2.1. Patient Characteristics and Clinical Signs

The study population and clinical signs were recently described [26]. Briefly, a total of 123 equine snakebite cases were included in the study. All incidences of envenomation occurred between April and November, within the snake habitat, and in an area where no other venomous snake species are found. Most horses were bitten in the face (73.4%) or legs (22.6%). Horses were of various breeds, but predominantly Arabians (42.7%), and Quarter Horses (19.4%). There were 59 males and 64 females. Ages ranged between 3 months and 23 years. The most common clinical signs associated with envenomation were local swelling (100%), elevated heart rate (66.7%), increased respiratory effort (61.6%), decreased appetite (50.5%), and hyperemic mucous membranes (45.7%).

### 2.2. Treatment

**Figure 1:**
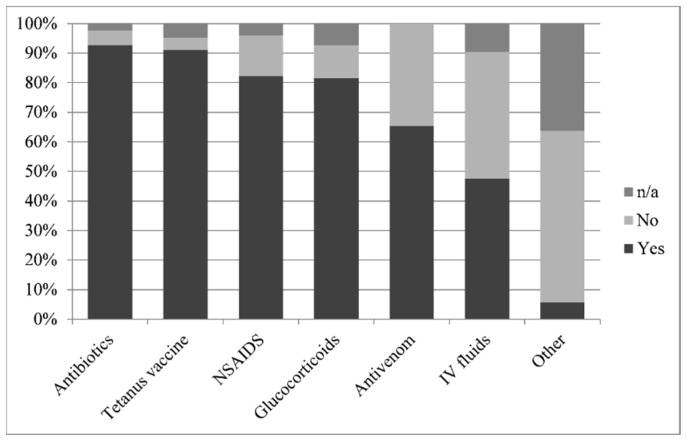
Frequencies of administration of different treatments following *Daboia palaestinae* envenomation displayed as percentage of 123 horses. [n/a = not available].

Although attended by 25 different veterinarians, most horses were treated with procaine-penicillin (92.7%), non-steroidal anti-inflammatory agents (NSAID), (flunixin meglumine or phenylbutazone, 82.3%), dexamethasone (81.4%) and tetanus toxoid [either by confirmation of recent vaccination (within 6 months) or vaccination on site, 91.1%]. Some horses received intravenous crystalloid fluids; 34 horses (27.4%) received less than 10 L and 25 horses (20.2%) received more than 10 L. A number of horses were administered antihistamines (diphenhydramine, four horses) or diuretics (furosemide, one horse) (Figure 1). One horse required a tracheostomy.

Since the majority of horses received most treatment components, it was difficult to isolate and successfully analyze the efficacy of any single agent. However, horses treated with procaine-penicillin were four times less likely to die [mortality rate of 4/6 (66.7%) untreated horses, as compared to 20/114 (17.5%) treated horses; reverse odds ratio (OR) = 9.4, 95% confidence interval (CI): 1.22–107.61, *p* = 0.014].

Eighty horses (65%) were treated with antivenom. Since antivenom is the only specific treatment for envenomation, the association between all other treatments and case fatality was re-evaluated in those cases in which the horse received no antivenom (*n* = 43). Only two horses in this group were not treated with procaine penicillin, and both died (in contrast to 9 out of 40 treated horses); however, this association was not statistically significant (*p* = 0.064).

**Figure 2:**
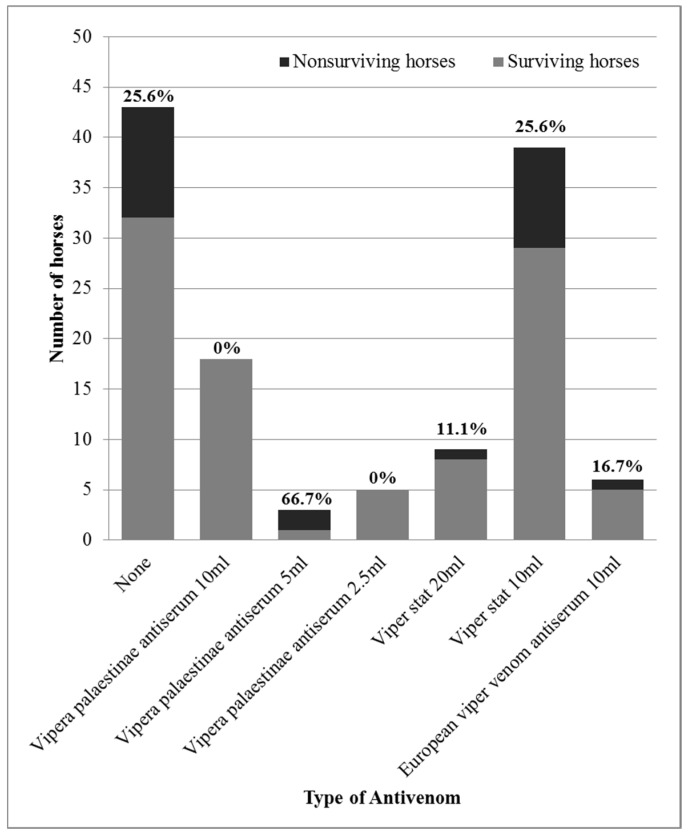
Mortality rates of horses treated with different products and dosages of antivenom following *Daboia palaestinae* envenomation.

The horses receiving antivenom were treated with different brands and varying dosages (Figure 2). Clinically relevant adverse reactions were not observed during the treatment in any of the horses. No significant association was found between the administration of antivenom and the administration of procaine-penicillin (*p* = 0.66). Therefore, it was concluded that penicillin treatment could not be a confounding variable in the association between antivenom administration and case fatality, despite the fact that penicillin treatment was also significantly associated with case fatality. Associations between antivenom administration and other treatments were not analyzed, since they were not associated with reduced mortality and were, therefore, ruled out as potential confounding variables.

### 2.3. Response to Treatment

**Figure 3:**
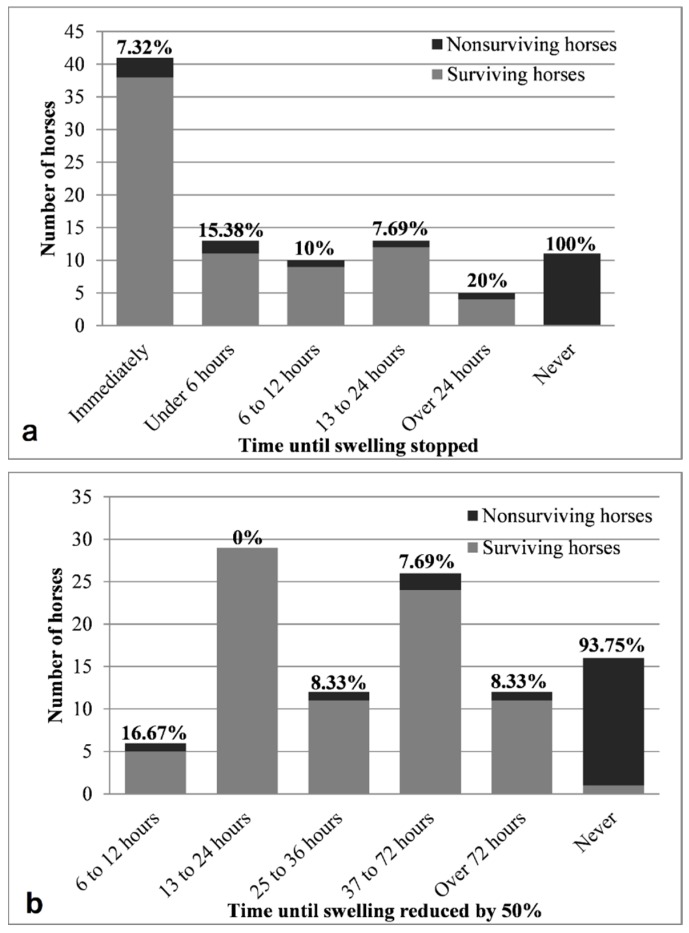
Mortality rates of horses treated for *Daboia palaestinae* envenomation, in relation to (**a**) cessation of progression of clinical signs (local swelling), and (**b**) improvement of clinical signs (50% reduction of local swelling), as subjectively assessed by the attending veterinarian. The percentage of fatal cases within each group appears in bold above each bar.

Response to treatment was subjectively evaluated by the 25 participating veterinarians and is described in Figure 3. Both cessation and reduction of local swelling had moderate linear correlations with case mortality rate (ρ = 0.464 and ρ = 0.549 respectively, *p* < 0.01). Almost all patients that exhibited no improvement following treatment did not survive (only 1/16 survived up to the end of the 4-week follow-up). Most (22/25) of the fatal cases died within 24 h of envenomation, while only three cases deteriorated and died between 1–4 weeks later [26]. The chance of fatality in cases that did not respond to treatment was over ten times that of cases that did respond to treatment at any point in time (OR = 202, 95% CI: 12–3319, *p* < 0.001). Although patients that responded immediately to treatment were 4.2 times less likely to die (OR = 0.18, 95% CI: 0.05–0.65, *p* = 0.008), this group did not differ statistically from the groups that responded at any other time (OR = 0.57, 95% CI: 0.13–2.51, *p* = 0.712). Horses that did not improve at all after treatment were 15.9 times more likely to die than horses that did improve at some point (OR = ∞, 95% CI: 17.8–∞, *p* < 0.001). Cases that improved within 24 h were 10 times less likely to die (OR = 0.07, 95% CI: 0–0.51, *p* < 0.001).

The time interval between the snakebite event and the administration of treatment was not significantly associated with the response to treatment (*p* = 0.339), clinical improvement (*p* = 0.558) or outcome (*p* = 0.439). No significant association was found between administration of antivenom and either response to treatment or swelling reduction (*p* = 0.177 and *p* = 0.658, respectively).

### 2.4. Determination of Antivenom Concentration in Two Commercially Available Antivenom Products

The potency of Viper-stat^®^ antivenom was found to be 27 PD_50_/mL (mice), which concurred with the manufacturer’s data (≥25 PD_50_/mL) and for *Vipera palaestinae* antiserum ≥100 PD_50_/mL, which also concurred with the manufacturer’s data (Appendix A Table A1).

### 2.5. Antivenom Administration as a Prognostic Factor

**Figure 4:**
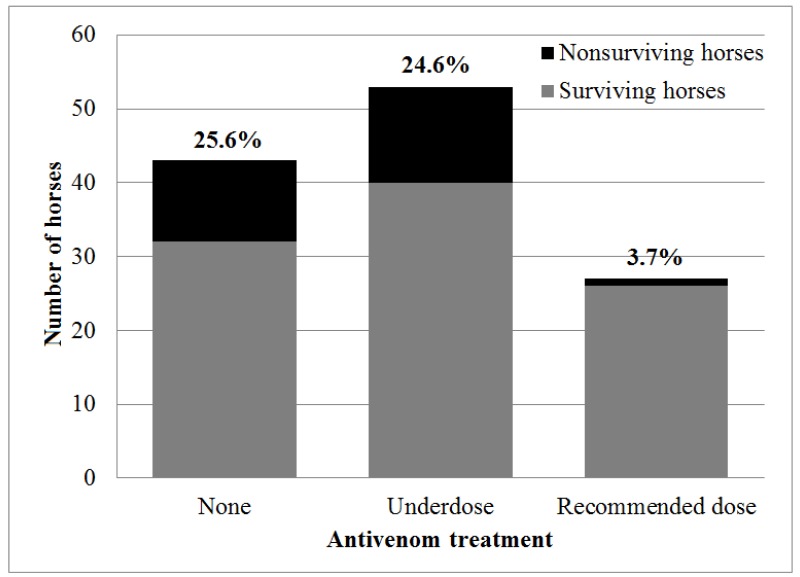
Mortality rates of horses treated with the recommended dosage of species-specific antivenom (10 mL/1000 PD_50_ *Vipera palaestinae* antiserum or 20 mL/500 PD_50_ Viper-stat^®^) in comparison to horses receiving no antivenom, or treated with less than the recommended dose (underdose) or with a non-specific antivenom product following *Daboia palaestinae* envenomation. The percentage of fatal cases within each group appears in bold above each bar.

The overall case fatality rate of horses with *D. palaestinae* envenomation was 20.3% (25/123 horses). The fatality rate did not differ between horses treated (14/80 horses, 17.5%) or not treated (11/43 horses, 25.6%) with antivenom (OR = 0.62, 95% CI: 0.23–1.69, *p* = 0.349). However, horses were treated with various products and dosages of antivenom due to cost and availability considerations (Figure 2). When comparing different products and dosages separately, a significant association was revealed. There was a decrease in mortality when treatment included one vial (10 mL/1000 PD_50_) of *Vipera palaestinae* antiserum (*p* = 0.047), leading to a decrease in mortality in comparison to untreated horses (OR = 0, 95% CI: 0–0.822, *p* = 0.025). Moreover, treatment with antivenom using the recommended dosage (10 mL/1000 PD_50_ of *Vipera palaestinae* antiserum, 10 mL of European viper venom antiserum or 20 mL/500 PD_50_ of Viper-stat^®^) was associated with decreased mortality (OR = 0.19 95% CI: 0.02–0.85, *p* = 0.021), and treatment with the recommended dosage of species-specific antivenom (excluding the European product) resulted in more significant results. There was a 3.7% (1/27 horses) mortality rate in horses treated with the recommended species-specific dosage, and they were 6.7 times less likely to die (3.7%, 1/27 horses, in comparison to 25%, 24/96 of the horses, OR = 0.12, 95% CI: 0–0.79, *p* = 0.014) (Figure 4).

Since the use of antimicrobials was also found to be a protective factor, the effectiveness of the use of antivenom was re-analyzed, while including horses treated with procaine-penicillin (*N* = 114). The associations between treatment with one vial (10 mL/1000 PD_50_) of *Vipera palaestinae* antiserum (*p* = 0.012), treatment with the recommended dose of any antivenom (*p* = 0.014) and treatment with the recommended dose of species-specific antivenom (*p* = 0.04) and decreased mortality remained statistically significant within this group. The analysis was not performed on horses that were not treated with antimicrobials, since the group was too small (*N* = 6). In a multivariable statistical analysis, both the use of procaine penicillin (*p* = 0.01) and species-specific antivenom in the recommended dose (*p* = 0.023) remained significantly associated with decreased mortality.

Previously, we constructed a prognostic snakebite severity scale (SSS) adjusted to horses [26] (Appendix B Table A2). Analysis of the efficacy of antivenom administration for horses with poor prognosis (SSS >= 2) showed a significant reduction in mortality when the recommended dosage of any of the species-specific products was administered (OR = 0.11, 95% CI: 0–0.95, *p* = 0.036). Less conclusive results were attained when comparing all antivenom products and all dosages (*p* = 0.09). Only one of the horses with a good prognosis (SSS < 2) died, and that single horse did not receive antivenom. However, the efficacy of antivenom administration in this group could not be established due to the lack of fatal cases.

## 3. Discussion

This is the first study which describes the treatment of horses that were envenomed by *D. palaestinae*, including the use of antivenom in two-thirds of the horses. Horses seem to be sensitive to *D. palaestinae* envenomation, with a relatively high mortality rate (20.3%, 95% CI: 13.6–28.5%) which is significantly higher than in humans (5.7%) and in dogs (4.0%), but similar to cats (22.2%) [5,8,26,27]. Therefore, any case of *D. palaestinae* envenomation in horses should be treated as an emergency. In addition, horses are especially prone to asphyxiation caused by head swelling, since they are unable to breathe through their mouths. Mortality due to rattlesnake envenomation is also much higher in horses than in dogs and humans. The reason, however, is unclear [28].

Treatment for *D. palaestinae* envenomation in horses is based on the recommended protocol for horses following snakebite [23] although some of its components, such as the use of corticosteroids and antimicrobials, are controversial in the treatment of humans and dogs. In this survey, most horses received similar treatment and roughly two-thirds (65%) received antivenom. Clearly the fact that the vast majority of horses (over 80%) were treated with antibiotics, NSAIDs and glucocorticoids makes it difficult to successfully analyze and interpret the efficacy of any single medication. However, in clinical settings, this cannot be prevented since treatment, including the use of antivenom, is determined by the clinicians, based on their clinical experience and the owner’s decision.

During the period of this survey, three antivenom products were used, which differ in availability and price. The main antivenom product used (48/80) was Viper-stat^®^, which is a polyvalent F(ab’)_2_ antivenom manufactured in Mexico for veterinary use. It comprises of a mixture of antivenom for snakes prevalent in North Africa and the Middle East. Since this was the first time, to our knowledge, that this product was used to treat *D. palaestinae* envenomation, we conducted a study on mice and found that the potency of this product, specifically against *D. palaestinae* venom, concurred with the manufacturer’s data. The recommended dosage for use in animals is 20 mL (two vials/500 PD_50_), which proved to be effective in decreasing mortality in horses in this study. This is the first veterinary study, to our knowledge, reporting on the clinical use of this product in animals. Studies evaluating a similar product intended for humans reported favorable outcomes with relatively few complications [29,30]. Adverse reactions were not reported in any of the horses, including those treated with Viper-stat^®^, thereby indicating its safety. Therefore, the results of this study support the use of this product as being safe and effective in horses and underscore the importance of administering at least two vials.

The second most-used product in this survey (26/80) was *Vipera palaestinae* antiserum. This product is manufactured in Israel using the venom of local snakes for the treatment of humans. The material used in the production comprises of a mixture of venoms extracted from snakes captured in different parts of Israel, in order to better represent the diversity of venom composition [4]. We also tested this product in mice and found that the PD_50_ for a single 10 mL dose is four times higher than the PD_50_ of the alternative Viper-stat^®^, as declared by the manufacturer. There is no “official” recommended dosage of this product for horses, since it has only been evaluated in human beings. In human medicine, the initial dose is usually 50 mL (five vials/5000 PD_50_), and additional treatment may be provided as necessary [2,13,15,16]. This “to effect” application is not feasible in horses, mainly for economic reasons, and one 10 mL dose was the previous standard treatment by equine clinicians. This dosage did prove to be effective and led to a significantly improved prognosis in comparison to other products and dosages, and in comparison to untreated horses. As in all other cases of antivenom administration in this study, clinically significant adverse effects were not observed following the use of this product.

It is difficult to draw unequivocal conclusions regarding the efficacy and safety of the third product, European viper venom antiserum, since it was only administered to six horses, although five of them did survive. The European viper venom antiserum that was administered to six horses in this survey is species-specific to *V. xantinae*, not to *D. palaestinae*. In-vivo studies in mice and horses showed that polyvalent antisera induce cross-protection to other viper venoms [31,32]. Specifically, pentavalent antisera with a similar composition to the one used here were shown to induce paraspecific activity against *D. palaestinae* venom [33]. Further studies are required to evaluate its effectiveness in the treatment of horses envenomed by *D. palaestinae*.

The use of a lower-than-recommended antivenom dosage did not prove beneficial for the treatment of horses in this study. The high, prohibitive price of *Vipera palaestinae* antiserum made it less accessible to horse owners, which resulted in the use of lower dosages and alternative products. The fact that the PD_50_ of the recommended dosage of Viper-stat^®^ (two vials/20 mL/500 PD_50_) is equivalent to half of the *Vipera palaestinae* antiserum 10 mL dose (one vial/1000 PD_50_) led some practitioners to administer 5 mL of the *Vipera palaestinae* antiserum (500 PD_50_) so as to lower the cost of treatment. Also attempting to save costs, only ¼ of the *Vipera palaestinae* antiserum dose (2.5 mL/250 PD_50_) was administered to several horses, since this is equivalent to one vial of Viper-stat^®^ (10 mL/250 PD_50_). However, the use of lower dosages of either product failed to curtail the mortality of envenomed horses and should be avoided.

Antivenom treatment proved to be efficacious even in cases with a poor prognosis. Treatment with the recommended dosage of species-specific antivenom for horses with severe clinical signs, i.e., scoring high on the recently-constructed horse-adapted SSS [26], still significantly reduced mortality. While the use of antivenom is advisable in these severe cases, it could not be evaluated in milder cases, where the mortality rate was low. In human medicine, the clinical severity of each envenomation affects the decision of antivenom administration and dosage [2,13,15,16,34]. Further research is needed to evaluate the recommended treatment dosage in horses with regard to the severity of clinical signs.

Adverse reactions to antivenom administration were not described in any of the treated horses. Although anaphylactoid and anaphylactic reactions are relatively common following antivenom administration in humans [2,13,15,17] and in dogs [35], a controlled study of whole IgG polyvalent antivenom administration to non-envenomed horses (although not specific to *D. palaestinae*) did not document any signs of anaphylaxis or serum sickness [36]. The absence of reported adverse reactions may reflect species differences, but it may also suggest that these symptoms in humans are caused by the introduction of foreign (equine) protein.

The use of antimicrobials following envenomation is controversial. In this study, even though 93% of horses were given antimicrobial prophylaxis using penicillin, its use was significantly associated with decreased mortality. Although routine antimicrobial use is not supported in cases of human envenomation [11,12,15], bacterial infection was one of the common complications described in *D. palaestinae* envenomed dogs [3]. Most of these infections were clostridial [3], making penicillin a good choice of treatment. This supports the recommendation of antimicrobial use in horses [23], though its efficacy should be further explored.

Treatment with anti-inflammatory drugs in snakebite patients is also controversial [23], and is not a part of the standard treatment of humans and dogs [3,5,11,13,27]. Anti-inflammatory drugs may help reduce inflammation, pain and swelling, but may also enhance the thrombocytopenia caused directly by the venom [23]. Corticosteroids may also, theoretically, interfere with antivenom activity. In human medicine, the use of corticosteroids is limited to cases of anaphylaxis [11], and some clinicians believe it is contraindicated [37]. In dogs, administration of corticosteroids has been associated with increased mortality following *D. palaestinae* snakebite [5]. In this study, over 80% of horses were treated with either NSAIDs or corticosteroids, and 69% of all horses were treated with both. The use of these drugs did not seem to affect case fatality, although the small number of untreated horses may have reduced the statistical power of the analyses, and a prospective randomized control trial is required to further investigate the efficacy and safety of these medications.

This four-year study entailed a large-scale survey of envenomed horses. Although the study was planned prospectively, there was no control or intervention in the treatment provided by the attending veterinarians. Despite this fact, most of the horses were treated with a limited number of medications and many received all treatment components. This was beneficial when assessing the effect of antivenom, since most horses, both in the treatment and control groups, received similar supportive care. Also, since in many cases the snakebite event was not witnessed, it was difficult to evaluate the time interval between the snakebite event and treatment. The importance of rapid treatment is well established in humans [2,12,13,16]. However, in this study, there was no apparent difference in the response to treatment in relation to the time of initial treatment, probably due to inaccuracy of the data. Another limitation of this study in the evaluation of the efficacy of antivenom was the variety of products and dosages, which decreased the statistical power of the analysis. In addition, since in most cases, the data were collected after the outcome was already known to the reporting veterinarian, the study was actually retrospective and, consequentially, contained potential bias.

This is the first study that evaluates the use of antivenom in the treatment of *D. palaestinae* envenomation in horses, and the results demonstrate that the use of species-specific antivenom in horses, in sufficient dosage, significantly improves patient prognosis. A major drawback in this analysis was the fact that several different products and varying dosages were administered; therefore, additional research is needed to support or refute our findings.

## 4. Conclusions

This research is part of an extensive study which evaluates the treatment of snake envenomation in horses. Species-specific antivenom and antimicrobial therapy proved to significantly reduce mortality in cases of *D. palaestinae* envenomation in horses and should be recommended for treatment protocol. Further research is needed to evaluate the appropriate antivenom dosage, as well as the efficacy of other commonly used medications.

## 5. Materials and Methods

### 5.1. Case Selection and Data Collection

Data regarding equine snakebite patients were collected from 25 experienced equine veterinary practitioners in Israel between April 2013 and August 2016. The participating practitioners were informed in advance and were asked to report to the authors, as soon as possible, every relevant case they encountered. In addition, all practitioners were called at least once a month to ascertain whether all equine snakebite cases had been reported. Only cases with history and/or clinical signs consistent with envenomation were included.

Veterinarians were asked to complete a questionnaire documenting each clinical case of envenomation. Detailed information describing the collected data, including the clinical signs at presentation and risk factors for mortality, have been recently described [26]. Briefly, details pertaining to the horse’s characteristics, snakebite event, local and systemic clinical signs, treatment, response to treatment, and outcome were collected. Final outcome (survival) was determined, in all cases, by a follow up four weeks post envenomation.

The study was approved by the Koret School of Veterinary Medicine Veterinary Teaching Hospital Internal Ethics Review Committee (KSVM-VTH/5_2013, 20 June 2013), and horse owners’ consent for inclusion in the study was obtained. 

### 5.2. Evaluation of the Response to Treatment

Three parameters were used to evaluate the response to treatment: the estimated time intervals between (1) the snakebite event and initiation of treatment, (2) administration of treatment and cessation of progression of local swelling, and (3) administration of treatment and 50% reduction in local swelling. These parameters are all estimations since, in many cases, the snakebite event was not directly observed and in the majority of cases, the estimated delay in response was documented by the owner. Therefore, these parameters were analyzed in categories.

### 5.3. Determination of Antivenom Concentration in Two Commercially Available Horse-Derived Antivenom Products

The experiments on mice were approved by the Ethics Committee of the Ministry of Health Central Laboratories.

Female ICR mice (*Mus musculus*), 18–20 g, were purchased from Harlan Laboratories, Inc. Lyophilized dry venom of *D. palaestinae* (manufactured according to GMP guidelines) was purchased from S.I.S Shulov Institute for Science LTD. In order to determine the LD_50_ of the venom and the PD_50_ of Viper-stat and *Vipera palaestinae* antiserum, we used an in-house method that is based on the original method of Reed and Muench, 1938 [38]. The lyophilized venom was weighed using analytical scales and divided into a single-use tube containing 10–15 milligrams of venom. The exact weight was written on each tube, and the tubes were stored at −30 °C in a desiccator until use. The venom was diluted in PBS to a stock solution of 100 microgram/mL that was further diluted according to Table 2, before IV injection into ICR mice. To verify the stability of the venom, we used a control chart for the LD_50_ results. The venom was found to be stable for at least 12 months without significantly losing potency.

For LD_50_ determination, groups of mice (6 per group) were injected intravenously (IV) with the following amounts of venom (in 0.5 mL phosphate buffer saline, PBS) 7.50, 8.75, 10.0, 11.25, 12.5, 13.75, 15.0, 16.25, 17.5, 18.75, 20.0 microgram venom/mice (Table 2). Twenty-four and 48 h post injection, the number of dead and live mice were recorded, and the LD_50_ was calculated according to the following formula (Equation (1)):

LD_50_ = X_2_ − [(P_2_ − 50) × (X_2_ − X_1_)]/(P_2_ − P_1_) (1)

X_2_ = the minimal amount of venom, which led to over 50% mortality.

X_1_ = the maximal amount of venom, which led to less than 50% mortality.

P_2_ = % of mortality when X_2_ was administered.

P_1_ = % of mortality when X_1_ was administered.

The PD_50_ is defined as the antivenom dosage that protects 50% of the animals challenged. The neutralization step was performed in vitro. Varying amounts of antivenom (diluted in PBS) were mixed with 5 × LD_50_ venom and incubated for 30 min at 37 °C. After incubation, the tubes were kept on ice until injection. Groups of 6 mice were injected IV with the antivenom that had been pre-incubated with 5 × LD_50_ (0.5 mL per mice). Twenty-four and 48 h post injection, the numbers of dead and live mice were recorded, and the PD_50_ was calculated according to the following formula (Equation (2)):

PD_50_ = X_2_ + [(P_2_ − 50) × (X_1_ − X_2_)]/(P_2_ − P_1_) (2)

X_2_ = the minimal amount of antivenom, which led to over 50% mortality.

X_1_ = the maximal amount of antivenom, which led to less than 50% mortality.

P_2_ = % of mortality when X_2_ was administered.

P_1_ = % of mortality when X_1_ was administered.

### 5.4. Statistical Analysis

Associations between response to treatment and case fatality were evaluated using χ^2^ or Fisher’s exact test, as deemed appropriate. Linear associations between scale parameters and mortality rates were evaluated using Spearman’s rho (ρ).

The association between antivenom treatment and fatality following envenomation was evaluated using χ^2^ test, and odds ratios were calculated with 95% confidence intervals. The association between administration of different treatment components was evaluated using χ^2^ or Fisher’s exact test, as deemed appropriate, to rule out potential confounders.

Since the three available products differ in the PD_50_ per dose, associations were calculated for each product and dosage separately, and also as dichotomous nominal parameters comparing treated versus untreated horses and horses treated with the recommended dose versus all other horses. The recommended dose was set at one vial (10 mL) of either *Vipera palaestinae* antiserum or European viper venom antiserum (since these products were never evaluated specifically for horses or veterinary usage) or two vials (20 mL) of Viper-stat^®^, which is a veterinary product. The “recommended dose” group was defined twice, once for all three products and again considering only the species-specific products (i.e., *Vipera palaestinae* antiserum and Viper-stat^®^). In addition, the association between antivenom administration and case fatality was assessed separately only for those cases that were treated with antimicrobials and once again for the cases with SSS equal or greater than the prognostic cutoff value of 2 [26]. This was done in order to evaluate antivenom efficacy, particularly in more severe cases.

Both treatments that were found significantly associated with deceased mortality (the use of antimicrobials and species-specific antivenom in the recommended dose) were included in a multivariable statistical analysis using a backward stepwise logistic regression model. Statistical significance was set at *p* < 0.05 for all analyses. The analyses were performed, using SPSS 22.0^®^ (IBM, Armonk, New York, US) and WinPepi 11.43^®^ (Abramson, J.H., Jerusalem, Israel) statistical software.

## Figures and Tables

**Table 1:** Antivenom characteristics of the products used to treat cases of *Daboia palaestinae* envenomation during the years 2013–2016.

Antivenom Characteristic	Kamada	Viper-Stat	European
**Product**	*Vipera palaestinae* antiserum	Viper-stat^®^	European viper venom antiserum
**Manufacturer**	Kamada Ltd., Israel	BioVeteria, Life Sciences, LLC, Mexico	Institute of Immunology, Croatia
**Type**	Whole Ig, monovalent	F(ab)_2_, polyvalent	F(ab)_2_, polyvalent
**Source**	Horse plasma	Horse plasma	Horse plasma
**Use**	Human and veterinary	Veterinary	Human and veterinary
**Active components in 10 mL dose**	1000 PD_50_ *Vipera palaestinea*	250 PD_50_ *Vipera paleestinea*	500 PD_50_ *Vipera xantinae*
**(As declared by the manufacturer)**		250 PD_50_ *Macrovipera lebetina obtuse*	1000 PD_50_ *Vipera ammodytes*
		250 PD_50_ *Naja palida*	1000 PD_50_ *Vipera aspis*
		250 PD_50_ *Walterinesia aegyptia*	500 PD_50_ *Vipera berus*
		500 PD_50_ *Bitis aretans*	500 PD_50_ *Vipera lebetinae*
		500 PD_50_ *Cerastes cerastes*	
		500 PD_50_ *Naja haja*	
		200 PD_50_ *Echis leucogaster*	
		200 PD_50_ *Macrovipera deserti*	
**Recommended starting dosage**	10 mL (50 mL for humans)	20 mL	10 mL

**Table 2:** Serial dilution of purified *D. palaestinae* venom for injection into mice to determine its LD_50_.

Tube Number	Dose of Venom per Mouse (µg/0.5 mL)	Venom Concentration (µg/mL)	Volume from the Venom Stock Solution (100 µg/mL)	PBS (mL)
1	5	10	0.4	3.6
2	6.25	12.5	0.5	3.5
3	7.5	15	0.6	3.4
4	8.75	17.5	0.7	3.3
5	10	20	0.8	3.2
6	11.25	22.5	0.9	3.1
7	12.5	25	1	3
8	13.75	27.5	1.1	2.9
9	15	30	1.2	2.8
10	16.25	32.5	1.3	2.7
11	17.5	35	1.4	2.6
12	18.75	37.5	1.5	2.5
13	20	40	1.6	2.4
14	21.25	42.5	1.7	2.3
15	22.5	45	1.8	2.2
16	23.75	47.5	1.9	2.1

## Appendix A

Determination of antivenom concentration in two commercially available products. First, establishment of the LD_50_ of the venom by injecting venom in serial dilution to mice (a). Second, determination of the PD_50_ of (b) Viper-stat and (c) *Vipera palaestinae* antiserum by injecting ×5 LD_50_ of venom mixed with serial dilutions of antivenom after incubation in 37 °C for 30 min. At each step, the number of living versus dead mice in each group was recorded, the values were added and ordered in assending order of the dead mice and in descending order of the live mice (cumulative); the middle value of this list marked the 50% mortality value from which the LD_50_ or PD_50_ were calculated.

**Table A1:** Determination of antivenom concentration in two products.

	Dose of per Mouse (µg)	Mice			Cumulative			Mortality (%)	
		N	Living	Dead	Living	Dead	Total		
**a**	**Venom**								
	8.75	6	2	4	17	4	21		
	10	6	5	1	15	5	20		
	11.25	6	4	2	10	7	17	41	P_1_
	12.5	6	4	2	6	9	15	60	P_2_
	13.75	6	2	4	2	13	15		
	LD_50_ = 12.5 − [(60 − 50)/(60 − 41) × (12.5 − 11.25)] = 11.84								
	LD_50_ = 42.23								
	LD_50_/mouse = 5.3								
**b**	**Viper-Stat**								
	0.1875	6	4	2	21	2	23		
	0.175	6	3	3	17	5	22		
	0.1625	6	6	6	14	11	25	44	P_1_
	0.15	6	4	2	8	13	21	62	P_2_
	0.1375	6	4	2	4	15	19		
	ED_50_ = 0.150 + [(62 – 50)/(62 − 44) × (0.1625 − 0.15)] = 0.1583 mL								
	1 mL antivenom = 27 LD_50_ = 0.32 g venom								
**c**	***Vipera palaestinae* Antiserum**								
	0.0312	6	4	2	12	2	14		
	0.025	4	2	2	8	4	12		
	0.0187	4	3	1	6	5	11	45.5	P_1_
	0.0124	4	3	1	3	6	9	66.66	P_2_
	ED_50_ = 0.0124 + [(66.65 − 50)/(66.65 − 45.5) × (0.0187 − 0.0124)] = 0.0174 mL								
	1 mL antivenom = 247 LD_50_ = 2.9 g venom								

## Appendix B

Snakebite Severity Scale is constructed in such a way as to assist equine practitioners in estimating patient prognosis following *D. palaestinea* envenomation. The seven items are totaled to derive a score between 0 and 7. Results of 2 or more points indicate a graver prognosis (sensitivity-95.5%, specificity-67.9%). [BAR = bright, alert and responsive; QAR = quiet, alert and responsive; D = depressed.]

**Table A2:** Snakebite Severity Scale calculation.

Clinical Parameter	0	1
**Weight (Kg)**	Over 200 kg	Under 200 kg
**Swelling (scale 1–5)**	1–3	4–5
**Respiratory effort (scale 1–5)**	1–4	5
**Mental status**	BAR or QAR	D or Shock
**Envenomation site**	Other	Hind leg
**Signs of bleeding disorder**	No	Yes
**Hydration status**	Under 7%	Over 7%
